# An update on leprosy immunopathogenesis: systematic review

**DOI:** 10.3389/fimmu.2024.1416177

**Published:** 2024-09-06

**Authors:** Marcos Jessé Abrahão Silva, Caroliny Soares Silva, Thiago Pinto Brasil, Ana Karoliny Alves, Everaldina Cordeiro dos Santos, Cristiane Cunha Frota, Karla Valéria Batista Lima, Luana Nepomuceno Gondim Costa Lima

**Affiliations:** ^1^ Postgraduate Program in Parasitic Biology in the Amazon (PPGBPA), University of Pará State (UEPA), Belém, Brazil; ^2^ Department of Biomedicine, Federal University of Ceará (UFC), Fortaleza, Brazil; ^3^ Bacteriology and Mycology Section (SABMI), Evandro Chagas Institute (IEC), Ananindeua, Brazil; ^4^ Department of Pathology and Legal Medicine, Faculty of Medicine, Federal University of Ceará (UFC), Fortaleza, Brazil

**Keywords:** leprosy, immunity, pathology, molecular, mycobacterium leprae, nerve damage

## Abstract

**Introduction:**

Leprosy is a chronic infectious condition and the main cause of neuropathy that occurs brought on by *M. leprae*. It is known that the biological characteristics of the human host, such as the immunological ones, have a higher influence on the pathology of this disease than the intrinsic mechanisms of the bacterium. The objective of this work was to review the scientific knowledge about the relationship between immunopathology and the severity of leprosy.

**Methods:**

A systematic review following the PRISMA 2020 recommendations was conducted in the PUBMED, LILACS, SciELO and Science Direct databases using articles in English, Portuguese or Spanish between January 2011 and May 2022 with the descriptors “Leprosy/Immunology”, “Cytokines” and “*Mycobacterium leprae*”. A methodological quality assessment was carried out using the JBI checklists.

**Results:**

A total of 49 articles were included. There is a relationship of greater severity of infection associated with lower release of MHC molecules in response to PGL-1 that inhibit the promotion of resolving T lymphocytes arising from dendritic cells (DCs) stimulation. In addition, the differentiation of macrophage phenotypes dependent on the activation of PRRs can define activation and the distinct type of T helper (Th) cells involved according to severity. Activated CD8+ T cells also have distinct types at the appropriate poles of the disease, and B cells show at the most severe pole of the LL, specific induction of IgA and more Treg-type CD8+ T cells that further contribute to T cell anergy.

**Conclusion:**

Therefore, the adaptive immune system aggravates nerve damage and defines the type of leprosy, while the innate immune system is considerably more significant in the onset of nerve damage, symptomatic of the initial presentation of illness and in several critical immune responses, including inflammation and elimination of dead *M. leprae*.

## Introduction

Leprosy or Hansen’s Disease (HD) is a chronic infectious condition and the main cause of infectious neuropathy caused by the obligate intracellular organisms *Mycobacterium leprae* (*M. leprae*) and *M. lepromatosis*. This mycobacteriosis is characterized by high infectivity and minimal pathogenicity (1). The epidermis and peripheral nerves are the primary targets of this micobacteria. Damage to the peripheral nervous system can result in sensory and motor loss, as well as hand and foot abnormalities (2).

The most common cause of leprosy in humans is *Mycobacterium leprae*. It affects people all over the world and, in 2019, over 200,000 new instances from more than 100 countries were reported, although around the globe, Brazil, India, and Indonesia account for roughly 80% of all newly reported cases (3). High endemicity regions can be observed inside nations with lower levels of development (4).

Physical impairment can be developed before leprosy diagnosis, throughout therapy, and after discharge from treatment (5). Leprosy-related disabilities affect about 15% of people worldwide (6). Disability and nerve thickening was already linked in leprosy (7). The peripheral nerves’ Schwann cells, which are crucial for transmitting nerve impulses, and dermal histiocytes (tissue macrophages) are the two main cell types that this bacterium primarily invades. In the form of erythematous infiltration, skin lesions manifest as whitish papules or rashes (8).

A wide range of clinical symptoms are brought on by delayed diagnosis and inadequate leprosy treatments. Due to its morbidity and socioeconomic effect, which are both results of sequelae (such as physical impairment and deformities) that arise during the clinical course of the illness, leprosy is regarded as a significant public health issue (9). Regarding the clinical, bacilloscopic, histological and immunological presentation, the Ridley-Jopling classification of leprosy has been divided into five types related to the order of increasing severity: tuberculoid (TT), borderline tuberculoid (BT), mid-borderline (BB), borderline lepromatous (BL), and lepromatous (LL) (10). In this sense, the clinical spectrum of the disease can be summarized in a variation of severity between the Tuberculoid and Lepromatous poles, being separated by Borderline forms in the middle (11).

People who have weak cell immune responses against the bacillus also have strong humoral immune reactions and large levels of serum antibodies, which makes it difficult for them to control the spread of *M. leprae*. While LL is characterized by robust humoral immunity, TT is linked with strong cellular immunity, poor humoral immunity, and granulomatous local skin lesions (12).

Individual differences in the clinical manifestation of the illness are mostly caused by unique host characteristics rather than genetic variants of the bacterium (13). So, this study sought to review the correlation between the immunological characteristics of the human host and the distinct clinical presentations of leprosy based on its severity poles (with no focus on leprosy reactions, such as reverse reaction - RR and type 2 leprosy reaction, also known as erythema nodosum leprosum – ENL forms, and the Lucio’s phenomenon – LP), caused by *M. leprae*. The aim of this article was to write an updated review on the immunopathogenesis of leprosy.

## Material and methods

### Study design

This work is a systematic review conducted in accordance with the Preferred Reporting Items for Systematic Reviews and Meta-Analyses (PRISMA) 2020 statement (14). To create the guiding question, the PICO strategy was used with the following anagrams: population, intervention, comparison, and outcome (15). In this context, it was developed from Population: patients with leprosy; Intervention: to assess immunopathogenic features in *Mycobacterium leprae*-infected patients; Comparison: mycobacteria’s immunopathogenic properties to human immunity; Outcome: the disease’s progression. So, the following question was formed: “Which immunopathological aspects of the host are associated with the types of clinical forms of leprosy?”.

### Search strategy

The descriptors ((“Leprosy/immunology”[Mesh]) AND “Cytokines”[Mesh]) AND “ *Mycobacterium leprae* “[Mesh] were combined to identify and select relevant articles from the databases Science Direct, the National Library of Medicine National Institutes of Health of the USA (PUBMED), Latin American and Caribbean Literature in Health Sciences (LILACS) and Scientific Electronic Library Online (SciELO). The period included the period from January 2011 to May 2022. The information was gathered on July 20, 2022. The study only considered English, Portuguese, and Spanish as languages. Study titles and abstracts were reviewed, and those that addressed this topic were given greater consideration for analysis. All of those published before 2011 were excluded, as well as those that were duplicated, only available as an abstract, letters to the editor, and papers with inaccessible key material.

### Extraction of data and quality methodological assessment

All relevant data, including any discrepancies and ambiguities discovered in the articles, were separately gathered, extracted, and reviewed for quality by two authors (MJAS and CSS), with the assistance of a third author (TPB) in situations where the selection of the data was discordant. The data extracted from the articles were: author and year of publication; data base; kind of study; goals; results. The data found were organized in Microsoft Office Excel 365 and arranged in tabular form.

The quality assessment was done by completing the Joanna Briggs Institute (JBI) Appraisal Checklists for the kind of studies included, such as: Checklist for Cohort studies (ranging from 0 to 11); Checklist for Systematic reviews and research syntheses (ranging from 0 to 11); Checklist for Quasi-experimental studies (ranging from 0 to 9) (16). The scores for answering the checklist questions were considered only when the conditioned answer was “Yes” (17).

## Results

### Literature search

**Figure 1:**
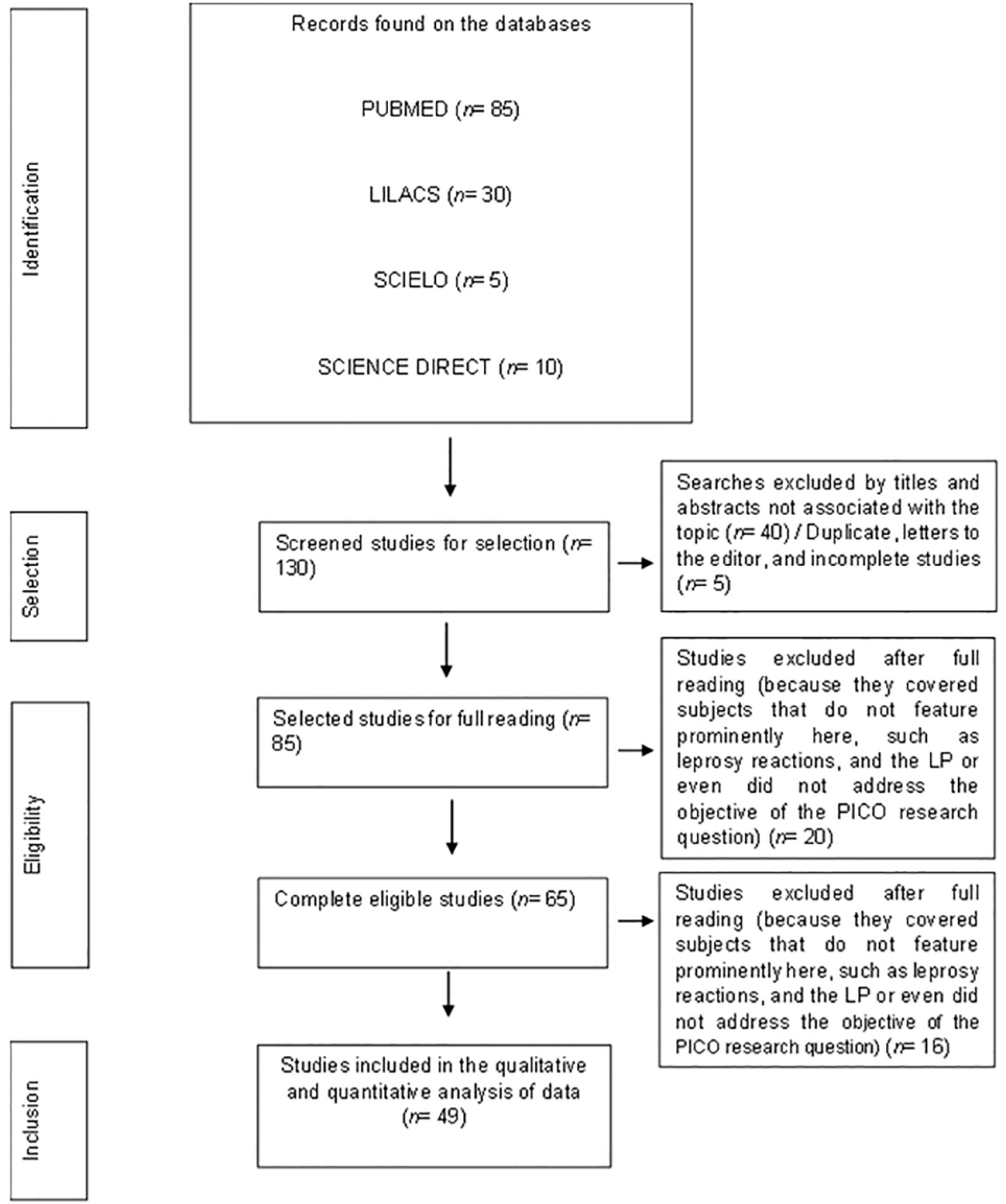
PRISMA flowchart of the steps related to the inclusion of articles in this systematic review.

A total of 130 studies were found in an initial search in the analyzed databases, being collected for the selection phase. During the selection, 5 incomplete or duplicated studies between the databases and 40 researches not relevant to the theme considering the reading of the abstract and title were excluded. So, 85 studies selected for reading in full were verified if they concerned the subject and then, 36 of them were excluded from the analysis due to eligibility. In the end, 49 studies were included for qualitative synthesis (**Figure 1**).

### Presentation of the data included in this review

The final framework consists of 49 papers. The articles included in this review were mostly international (n= 32; 65%), in the English language in its entirety (n= 49; 100%), coming from PUBMED (n= 49; 100%) and referring to the type of cohort study (n= 25; 51%), experimental (n= 16; 33%) and review (n= 8; 16%). The methodological quality assessment score demonstrated low bias in these data. These extracted data were arranged in tabular form below (**Table 1**).

**Table 1:** Characteristics of the articles included in this systematic review.

Author and year of publication [Reference]/Database/Type of Study/JBI Score	Objectives	Results
FROES; SOTTO; TRINDADE (2022) (18)/PUBMED/Review/JBI score 11/11	Review current knowledge of cytokines, Th subpopulations and regulatory T cells that participate in each clinical presentation of leprosy.	Macrophages can be subdivided into M1 (associated with the Th1 response) related to the tuberculoid form, and M2 (associated with Th2) related to the lepromatous form. In addition to having already described M4 types of these cells related to the lepromatous form. These cells show high NOS and ROS release, while higher expression of the enzyme nitric oxide synthase (iNOS) has been described in cutaneous tuberculoid lesions, compared to lepromatous lesions, which can be attributed to the higher Th1 response. An increase in the count of dendritic cells (DCs) was observed in the tuberculoid pole, evidencing its protective role against the bacillus. Th25 cells have also been described in leprosy. Furthermore, Th22 are part of a group of CD4+ T cells that produce isoforms of the fibroblast growth factor (FGF) family and cytokines such as IL-22, TNF-α, IL-13 and IL-26. Regarding the B-cell response, disseminated disease was associated with increased amounts of IL-5 and IgM in the lesions, as well as increased expression of several B-cell gene pathways and functional groups. These findings are in line with the proximity of Lepromatous leprosy to the Th2 pole.
KARAMOVA et al. (2020) (19)/PUBMED/Experimental/JBI score 8/9	To comparatively analyze mice for modeling leprosy infection caused by several strains of *M. leprae* obtained from leprosy patients from different regions of Russia.	Immunodeficient albino animals (BALB/c) that have been infected by *Mycobacterium leprae* form a T-cell immune response with predominant involvement of Th1 and Th17 cells with significant induction of IL-2 and IL-17, creating advantages for the development of intracellular bacterial pathogens. BALB/c nude mice are characterized by the suppression of the adaptive immune response, being used preferentially in the reproduction of leprosy.
DOZ-DWBLAUWE et al. (2019) (20)/PUBMED/Experimental/JBI score 9/9	To investigate in parallel in macrophages, dendritic cells (DCs) and polymorphonuclear neutrophils (PMNs) to understand the impact of PGL-1 on the innate immune response.	The lipid virulence factor PGL-1 confers to *M. leprae* and to recombinant BCG the unique ability to activate CR3 for phagocytosis in three major subsets of dendritic cells (DCs), polymorphonuclear neutrophils (PMNs) and macrophages (MPs). Phagocytosis results in Syk-dependent cNFAT translocation to the nucleus which rejoins cells producing a cNFAT-specific signature of soluble mediators such as IL-2 by DCs, IL-10 by PMNs, IL-1β by MPs, and PGE2 by all three cells cited. Dectin-1 deficiency in DCs, PMNs and MPs resulted in reduced BCG phagocytosis, being independent of PGL. PGLs modulate infectivity by binding to the C-type Lectin Receptor (CR) 3, a complement system receptor.
SILVA et al. (2019) (21)/PUBMED/Cut-off study/JBI score 10/11	Identify the subsets of cytokine-producing T cells (IFN-γ, TNF, IL-10), the expression of their transcription factors.	Patients with lepromatous leprosy, whether reactional or not, had a CD4/CD8 frequency of 2:1. However, in healthy volunteers (HVs) there was a 1:1 ratio of CD4/CD8, in addition to the change in the frequency of whole T cells, after *in vitro* stimulation by *M. leprae*. This same group showed an increased frequency of CD4 and CD8 + T lymphocytes, although the CD4/CD8 ratio did not change.
MONTOYA et al. (2019) (22)/PUBMED/Cohort/JBI score 10/11	Investigate host-pathogen interaction at the site of human infectious disease.	The readings of *M.* leprae were abundantly detected in L*-*lep lesions. A significant correlation of *M. leprae* abundance with the IFN-β activation gene expression signature was detected, but no IFN-γ signature. The fraction of bacterial transcripts, which reflects bacterial burden, correlates with a type I interferon (IFN-I) gene signature known to inhibit antimicrobial responses. The mRNA to rRNA ratio, which indicates bacterial viability, is associated with bacterial heat shock proteins and the host B cell-activating factor (BAFF)-B cell maturation antigen (BCMA) antibody response pathway.
DANG et al. (2019) (23)/PUBMED/Experimental/JBI score 9/9	Verify how langerhans cells (LC) can exert an antimicrobial response with the ability to process bacterial-derived antigens for T cell presentation.	Treatment of Langerhans cells with interferon-gamma (IFN-γ) resulted in the activation of an antimicrobial activity, which depends on the upregulation of the antimicrobial peptide cathelicidin and the induction of autophagy. Induction of autophagy by IFN-γ promoted the fusion of phagosomes containing *Mycobacterium leprae* with lysosomes, facilitating the delivery of cathelicidin to the intracellular compartment where the pathogen is present. IFN-γ is also responsible for inducing vitamin D-dependent production of cathelicidin in Langerhans Cells, which is utilized in the antimicrobial response. Autophagy increased the ability of *M. leprae*-infected Langerhans cells to present antigen to CD1a-restricted T cells. The frequency of Langerhans cells presenting both cathelicidin and autophagic vesicles was higher in self-limited lesions compared with progressive lesions, indicating a correlation with the effectiveness of host defense against infection.
VILANI-MORENO et al. (2019) (24)/PUBMED/Experimental/JBI score 8/9	Evaluate the immune response and characterize the role of macrophages in immunocompetent and immunocompromised mice at different stages of the infectious process.	In nude mice, the infection was progressive, with bacterial multiplication, development of macroscopic lesions on the paws, and presence of bacilli in the popliteal lymph nodes after 8 months. In BALB/c mice, bacterial multiplication reached a plateau at 5 months and regressed at 8 months. Analysis of the paws revealed a mononuclear inflammatory infiltrate with a high number of neutrophils at 5 months, especially in nude mice. At 8 months, the number of neutrophils decreased, and the infiltrate became predominantly mononuclear in both mouse strains. There was no production of H_2_O_2_, O_2_^-^, IL-2, IL-4, IL-10, and IFN-γ in nude mice during infection. In contrast, BALB/c mice showed increased O_2_^-^ and IL-12 production at 5 months, and NO, IFN-γ, and TNF increased at 8 months, when bacterial load decreased. Anti-PGL-I antibody levels were higher in BALB/c mice.
QUEIROZ et al. (2019) (25)/PUBMED/Cohort/JBI score 11/11	To compare index response cases and their household contacts to identify immune response biomarkers in leprosy that may provide reliable evidence of subclinical infection in household contacts.	High levels of IgM and the highest degree of activation of CD4+ and CD8+ T lymphocytes in the index cases were detected. In addition, immunoglobulin levels of the anti-IgM antigens NDO-HSA, LID-1 and NDOLID were detected more in index cases than in household contacts. Greater activation of T lymphocytes was detected in index cases. Furthermore, in index cases, the phenotypic profile was more activated in CD4+ and CD8+ T lymphocytes. This activation was associated with a response to antigenic stimulation by the bacillus, generating an increase in CD86 expression by monocytes responsible for activating lymphocytes, generating effector or memory T cells. In addition, a lower frequency of CD4+ T lymphocytes was detected in the peripheral blood of the index cases.
ANDRADE et al. (2019) (26)/PUBMED/Experimental/JBI score 9/9	To characterize host-pathogen interactions based on an analysis of the *M. leprae* host transcriptome.	*M. leprae* infection results in a significant induction of the *NUPR1* gene, which has been identified as an IFN-β-specific gene in the *M. leprae* gene signature. *NUPR1* expression was highly induced by IFN-β, with a mean increase of 2.73-fold, whereas induction by IFN-γ was modest (0.42-fold). *NUPR1* induction in monocyte-derived macrophages (MDMs) after *M. leprae* infection was dependent on the IFN-β signal. The use of IFN-α receptor (αIFNAR) blocking antibodies demonstrated a dramatic reduction in *NUPR1* expression, indicating that IFN-β receptor activation is crucial for this induction. Bioinformatic analysis revealed that *NUPR1* is an upstream regulator of the gene signature induced by *M. leprae*, and this signature overlaps with that induced by IFN-β, suggesting a central role for *NUPR1* in the interferon-mediated immune response. NUPR1 overexpression was confirmed in progressive leprosy (L-lep) lesions by RT-qPCR and immunohistochemistry in skin biopsy samples, corroborating the relationship between IFN-β-induced gene expression and disease pathogenesis.
RASTOGI et al. (2019) (27)/PUBMED/Experimental/JBI score 7/9	Characterize ML1899 and identify its role in modulating the host immune response in the THP-1 cell line.	Analysis of the structure of ML1899 showed the presence of features of the α/β hydrolase-fold gene family. For the analysis of macrophage viability, the increase in ML1899 concentration after 10 µg/ml^-1^ directly involved cell reduction. Treatment of cells with LPS and ML1899 increased ROS (reactive oxygen species) production in the THP-1 cell line to 1.9-fold and 1.7-fold. ML1899 resulted in increased NO secretion in THP-1 cells, however it was lower than that of LPS. The presence of IgG against the ML1899 protein was verified in patients newly diagnosed with leprosy.
SALES et al. (2011) (28)/PUBMED/Experimental/JBI score 9/9	To analyze whether in chronic infections, effective immunity could be impaired by generation of IDO-mediated suppression (Indoleamine 2, 3-dioxygenase).	The number of IDO-positive cells was higher in LL than in BT lesions, where only positive cells scattered in the periphery and subepidermal area outside the granuloma were identified. In LL cells, there was a high positivity in the LL cellular infiltrate in which foam cells predominate, where they were found in isolated IDO+ dermal cells with dendritic morphology and in endothelial cells. Immunoblotting results showed that IDO expression was higher in LL biopsies than in BT. IDO activity was measured in patient serum, and it was found that IDO increased greatly in serum from LL patients. Also in patients’ serum, it was detected that serum levels of IFN-γ were higher in BT. *M. leprae* increased IDO activity in stimulated monocyte supernatants. In naive monocytes, infection increased IDO activity to levels approaching levels stimulated by IFN-γ. Immunoblot results showed that in LL monocytes, *M. leprae* and IFN-γ increased IDO protein synthesis.
KUMAR et al. (2011) (29)/PUBMED/Cohort/JBI score 11/11	Trace the parallel relationship, if any, between TGF-β, CTLA4, and Cbl-b.	The *CCR5*, *IFN-γ*, *IL-12B*, *IL-12RB2*, *IL-18*, *IL-18R1*, *IL-2*, *STAT1* and *TNF* genes were significantly decreased from BT/TT poles to BL/LL. Other Th1 genes also showed reduction, but without statistical significance. The *IL-2RA* (CD25) gene showed upregulation for BL/LL, showing the presence of an immunosuppressive environment by Treg cells. Downregulation was detected in key Th2 regulatory genes in relation to the BB/LL poles in leprosy: *CCL11*, *CCL5/RANTES*, *CCR3*, *CEBPB*, *NFATC2IP*, *GFI1*, *GPR44*, *IL-10*, *IL-13*, *IL-4*, *IL-5*, *JAK1*, *MAF*, *NFATC1* and *NFATC2*, where *TMED1* and *PCGF2* (Polycomb group ring finger 2) had maximum negative regulation, both in BL/LL and BT/TT. Weak upregulation of GATA-3 and increased expression of IL-10, relative to BL/LL, showed suppression of Th2 immune responses.
SAMPAIO et al. (2012) (30)/PUBMED/Cohort/JBI score 10/11	Identify, cytokine levels, biomarkers that could potentially discriminate the PB leprosy immune response and risk contacts.	The study measured the production of several cytokines, including IFN-γ, IL-2, IL-4, IL-10, IL-12p70, and TNF-α, in whole blood samples after stimulation with *M. leprae*-specific antigens. The analysis was performed in patients with paucibacillary (PB) and multibacillary (MB) leprosy, as well as in healthy contacts. The results showed that IFN-γ is the best indicator of antigen-specific cellular immune responses in patients with tuberculoid (TT) and borderline tuberculoid (BT) leprosy. However, it was not possible to effectively differentiate leprosy patients from healthy contacts based on the cytokines analyzed. Although antigen-specific responses in healthy contacts were qualitatively similar to those observed in TT/BT patients, some healthy contacts showed responses that more closely resembled those of patients with multibacillary (BL/LL) leprosy. This suggests that certain contacts may be at increased risk of developing the disease.
MOURA et al. (2012) (31)/PUBMED/Cohort/JBI score 10/11	lepromatous lesions exhibiting high CD163 and IDO expression linked to foamy appearance and iron deposits.	The study revealed that macrophages from patients with lepromatous leprosy have a regulatory phenotype, with high expression of CD163, which is regulated by IL-10. The presence of CD163+IDO+ cells was observed in the skin lesions and in the inflammatory infiltrate of patients with lepromatous leprosy. During the culture of macrophages isolated from lepromatous lesions, there was a gradual decrease in the gene expression of CD163, IDO and IL-10, as well as of the surface receptors CD163, CD209, HLA-DR, CD86 and CD14. This reduction coincided with the emission of *M. leprae* from these cells. Monocytes from healthy individuals, stimulated with irradiated *M. leprae*, showed an increase in the expression of IDO+CD163+CD209+ cells. Addition of cytochalasin B reduced CD163 expression, and the presence of anti-CD163 antibodies decreased bacterial entry into monocytes. Serum levels of sCD163, IL-10, and heme were elevated in lepromatous patients compared with tuberculoid patients and healthy controls. In addition, intracellular iron stores were also increased.
DAGUR et al. (2012) (32)/PUBMED/Experimental/JBI score 9/9	Decipher the mechanism of Man-LAM and PGL-1, the lipid components, in signaling events leading to T cell activation.	It was found that standardized doses of mannose-coated lipoarabinomannan (Man-Lam) and PGL-1 have been found to have log phase stimulation indices (SI) in both patients and healthy subjects. In healthy patients, a significantly lower level of SI with antigens was detected. T-cell Receptor (TCR)-triggered phosphorylation of Zap-70 was inhibited by Man-LAM and PGL-1, being evident in the presence of co-stimulation by CD28. Furthermore, it was noted that exogenous calcium influx was significantly attenuated by Man-LAM and PGL-1. The results also showed a decrease in cytosolic Protein Kinase C (PKC) activity in response to TCR or with CD28 stimulation, indicating a signal of cellular activation.
CHUNG et al. (2013) (33)/PUBMED/Cohort/JBI score 11/11	To investigate the potential role of galectin-3 in cell-mediated immunity using peripheral blood monocytes.	Galectin-3 expression was detected in the granulomatous infiltrate of LL patients, with 80-90% of strongly stained cells. In leprosy-T lesions only approximately 10% of the cells were weakly positive for galectin-3. Despite this increase, it has been identified that live *M. leprae* infection did not cause increased expression of galectin-3 messenger RNA or protein in human monocytes. Galectin-3 increased IL-10 release by 5.7 times compared to TLR2/1 stimulation. However, TLR2/1-induced IL-12p40 release was not affected. The addition of galectin-3 influenced the increase in the IL-10/IL12p40 ratio induced by TLR2/1 in monocytes. Lactose was able to inhibit the effect of galectin-3 on TLR2/1-induced IL-10 production. Galectin-3 caused >50% reduction of CD1b+ in GM-CSF-induced dendritic cells. Its ability to change the major histocompatibility complex (MHC) class II was also analyzed and it was seen that it did not have changes. Also in this case, the binding of lactose partially reversed the effect of galectin on these bindings.
TELES et al. (2013) (34)/PUBMED/Experimental/JBI score 8/9	Gene expression analysis of skin lesions of leprosy lesions.	It was indicated that IFN-β-induced genes, including IL-10, were significantly enriched in the profile. expression of the L-lep gene. Analysis of the scores showed that the IFN-β profile is higher and the IFN-γ profile is lower in L-lep lesions. Gene expression results also showed the differential expression in tuberculoid leprosy lesions (T-lep) lesions of the CYP27B1 mRNA, responsible for encoding vitamin D-1α-hydroxylase, and the mRNA for the vitamin D receptor (VDR). The ability of IFN-γ to upregulate CYP27B1 and VDR expression was blocked by the addition of IFN-β or IL-10, and this ability was reversed with the use of neutralizing monoclonal anti-IL-10. IL-10 also inhibited IFN-γ induction of CYP27B1 activity in macrophages. Ultimately, it was concluded that IL-10 and IL-4 coordinate to regulate vitamin D metabolism and catabolism, inhibiting IFN-γ-induced antimicrobial responses. The addition of anti-IL-10 and IFN-β reversed the ability of IFN-γ to upregulate the gene expression of the antimicrobial peptide, cathelicidin and DEFB4. IFN-γ also induces antimicrobial activity against *M. leprae* in monocytes and is blocked by pharmacological inhibition of the VDR, being completely annulled by the addition of IFN-β or IL-10 and reversed with the use of anti-IL-10.
KUMAR et al. (2013) (35)/PUBMED/Cohort/JBI score 11/11	To investigate DC SIGN-mediated IL-10 production and the role of histone acetyltransferases (HATs), p300 and activation of CBP-associated genes including the interaction of p300 at the IL-10 locus during *M. leprae* infections.	Analysis of cells treated with GM-CSF+IL-4 and GM-CSF+IL-4+ManLAM, the expression of the CD83 band was significantly higher than in untreated cells. In monocytes treated with GM-CSF+IL-4 and GM-CSF+IL-4+ManLAM, it was verified that a greater expression of DC SIGN than in untreated samples, as well as influenced the increase in IL-10 production. Higher NF-K β expression was observed in BL/LL patients, in addition to higher detection in DCs derived from monocytes treated with ManLAM from BL/LL compared to cells treated with DC SIGN and anti-DCSIGN+ManLAM. ^3^ H thymidine incorporation was noted in cases of ManLAM-treated PBMCs from BL/LL patients, in addition to an increase in ^3^ H thymidine incorporation when cells were treated with anti-IL-10.
KUMAR et al. (2014) (36)/PUBMED/Cohort/JBI score 11/11	Investigate the role of FoxP3 as a transcriptional repressor and/or activator.	Significant increases in FoxP3, HDAC7 and HDAC9 transcription levels were detected in CD4+ CD25+ cells in individuals with leprosy, showing the ability of HDCAC7/9 to act in FoxP3-mediated gene repression. High levels of FoxP3-HDAC7 and FoxP3-HDAC9 complexes were detected in CD4+ CD25+ cells derived from BL/LL patients, evidencing that FoxP3 is carrying HDAC7/9 to specific gene loci, being repressed under conditions induced by *M. leprae*, which later lead to overexpression of FoxP3. It was also revealed that FoxP3 acts as a transcriptional activator in CD4+ CD25+ Treg cells. In addition, FoxP3-HDAC7&9 and FoxP3.p3300 act on a molecular set within CD4+CD25+ cells, regulating immunosuppressive activities.
SAINI; RAMESH; NATH (2014) (37)/PUBMED/Cohort/JBI score 10/11	To investigate the role of Tregs in skin lesions and *in vitro* stimulated PBMC cultures of patients with lepromatous and tuberculoid leprosy.	*FOXP3, TGF-β* and *IL-10* was observed in lepromatous compared to tuberculoid. Flow cytometry analysis showed an increase in CD4+CD25+FOXP3+ cells in unstimulated basal cultures, indicating the presence of thymus Treg (tTreg) natural or *in vivo* induced Tregs during the disease. The results showed an increase in lepromatous, compared to tuberculoid, FOXP3 and TGF-β in populations of CD4+ CD25+ T cells. Treg cell population analyzed showed characteristics of natural and iTreg populations.
OLIVEIRA et al. (2014) (38)/PUBMED/Longitudinal cohort/JBI score 11/11	Evaluate the use of different combinations of *M. leprae* antigens to maximize the proportion of PB patients detected from the amount of IFN-y.	Results indicated that 3 out of 5 antigen combinations used to stimulate WBA (whole blood analysis) were able to increase levels of IFN-γ production in responders in the PB group and among contacts. This combination may have increased the availability of epitopes for recognition by effector and memory T cells. All patients with MB leprosy were negative for anti -PGL-1 and most had a positive WBA IFN-y response. MCP-1/CCL2, MIP-1β/CCL4 and IL-1β chemokines were significantly increased in leprosy patients and HHC compared to endemic controls. IFN-y levels produced by asymptomatic individuals were higher than in individuals with PB leprosy.
BOBOSHA et al. (2014) (39)/PUBMED/Cohort/JBI score 9/11	To investigate the Treg cells in *M. leprae* unresponsiveness in LL patients.	The production of IFN-y was reversed with the addition of CD25+, characterizing that the CD25+ cell population confers the lack of response in LL patients. The frequency of CD4+CD25+ FoxP3+ cells was higher in LL than in BT, but without statistical significance. As for CD8+CD25+FoxP3+ cells, the results showed a significantly higher frequency in LL, indicating a role in the LL response. Finally, significantly more CD68+ CD163+ were detected in the vicinity of FoxP3 cells in LL lesions compared to TT/BT lesions.
HAGGE et al. (2014) (40)/PUBMED/Experimental/JBI score 8/9	To analyze whether IL-10 deficiency leads to a more robust immune response against *M. leprae.*	The absence of IL-10, combined with NOS2 deficiency or even alone, does not provide a restriction to growth in the B6 resistant strain. Infection of 10NOS2-/-FP with *M. leprae* resulted in the accumulation of *M. leprae-*responsive CD4+ and CD8+ T cells in the granuloma and there was an infiltration of CD4+ T cells in the local nerves.
NATH et al. (2015) (41)/PUBMED/Review/JBI score 11/11	Identify the main methods of diagnosing leprosy.	TLR2 and 4 act in the recognition of the leprosy bacillus, activate monocytes and release IL-12, the cytokine responsible for the death of the bacillus. IFN-Y GM-CSF helps to increase the expression of TLR1, leading to inflammation through the production of TNF-α. IL-10 induces a phagocytic pathway and IL-15 induces an antimicrobial vitamin D pathway and it reduces phagocytosis. Lep patients have undetectable antibody and T-cell responses while L-lep patients have abundant antibodies and low T-cell responses. Studies have shown that Th17 cells were more associated with T-lep in skin lesions and in antigen-induced PBMC cultures. There was a greater association of Th17 with non-polarized Th0 types, suggesting that these cells may participate in a third type of Th in leprosy. In reactive states, the lesions show an increase in CD4+ and IL17+ production. Th1 and Th2 cytokines are expressed in T-lep and L-lep granulomas, respectively.
LYRIO et al. (2015) (42)/PUBMED/Experimental/JBI score 9/9	Understand the importance of skin and keratinocyte invasion by *M. leprae in vitro* in innate immunity during infection.	Analyzes showed that 7.3% of keratinocytes spontaneously expressed CD209, an *M. leprae* receptor on macrophages. Subsequently, a trend towards a decrease in CD209 expression on the surface of keratinocytes was shown when *M. leprae* was used as a stimulus and increased its expression on the surface of macrophages. A higher expression of CD80, T-cell costimulator, was detected in DC compared to macrophages and keratinocytes. In addition, *M. leprae* induces a significant increase in CD80 expression in macrophages, while in keratinocytes there was a tendency to decrease.
BRAGA et al. (2015) (43)/PUBMED/Retrospective cohort/JBI score 10/11	Understanding anergy in cell-mediated immunity in leprosy type LL compared to TT and HC patients.	The *in vitro* differentiation of monocytes (MOs) into DCs, as shown by CD11c, DC-SIGN and CD1a expression, was similar in leprosy patients and in healthy patients. There was an increased expression of HLA-DR, CD40, CD80 and CD86 in DCs after maturation. However, CD83 was the most important marker in this maturation. It was shown that IL-12p70 production increased considerably in healthy patients and TT patients after CD40 binding, with this increase being smaller in LL patients. TNF and IL-12p40 concentrations were higher in BL DCs when compared to iDCs in leprosy patients and healthy subjects.
AARÃO et al. (2016) (44)/PUBMED/Cohort/JBI score 11/11	Evaluate the expression of IL-17, NGF and NGRF in the different clinical forms of leprosy.	IL-17 was more expressed in lymphocytes in the granuloma. For NGF and NGF-R immunoexpression was detected in granulomas and dermal nerve fibers. The quantitative analysis of IL-17 from immunostaining showed the difference between the clinical forms of leprosy, with a greater participation in the TT form of the disease.
SADHU et al. (2016) (45)/PUBMED/Cohort/JBI score 10/11	Investigate how Treg cells influence other effector T cells and their relationship with the polarized state of cytokines in leprosy patients.	As for the regularity of Treg cells, the research identified a greater than fivefold increase in cases of lepromatous leprosy (BL/LL) than in cases of tuberculoid leprosy (BT/TT) and healthy contacts (HCs). Treg cells express high amounts of IL-10 BL/LL, demonstrating that these cells have a suppressor character. A greater regularity of Treg CCR4+ cells was verified in BL/LL people, when compared to BT/TT people. The research identified the expression of this receptor in Th17 cells in BT/TT cases as opposed to BL/LL. The study, when comparing IL-10+ Treg cells and IL-17+ helper T cells, identified a considerable inverse relationship in cases of BL/LL, this is indicative that stability in the path of Treg cells can lead to the cellular anergy seen in this case of patient. The same relationship was not considerable in cases of BT/TT. Blockade of IL-10 and TGF-β cytokines, performed alone or together, resulted in the release of T cells IL-17+ in cases of BL/LL. The existence of Th17-inducing cytokines causes a decrease in T-reg cells, however, this decrease was not verified when it occurred only the stimulus with TGF-β.
SCHENK et al. (2016) (46)/PUBMED/Experimental/JBI score 7/9	To investigate the effect of MDP structure on the innate immune response.	The identification of the wall dipeptide (MDP), present in the cell wall of mycobacteria, by NOD2, leads to the stimulation of inflammatory responses to deal with bacterial infection, such as the expression of IL-32. In people with leprosy, this cytokine acts in the defense against *M. leprae.* It indicates that *in vivo*, the MDP structure of *M. leprae* does not significantly influence the activation of immunity. *M. leprae* MDP, in addition to IL-32, it also induced the expression of IL-1β, IL-6, TNF-α and IL-12p40 by monocytes, as well as acting in the differentiation of dendritic cells.
POLYCARPOU et al. (2016) (47)/PUBMED/Cohort/JBI score 10/11	To investigate the possible involvement of TLR4 in *M. leprae* infection.	It has been identified in *M. leprae* the presence of a TLR4 activating ligand that brings about signal transduction. *Ex-vivo-*derived human macrophages possess TLR4. By neutralizing TLR4 with a monoclonal antibody, with subsequent incubation in the presence of dead Hansen’s bacillus, a consistent reduction in the pro-inflammatory cytokines TNF-α, IL-6 and CXCL10/IP-10 was observed.
DE SOUSA et al. (2017) (48)/PUBMED/Cohort/JBI score 1011	Evaluate the cytokine profile of Th9 cells in disease.	Th9 cells produce IL-9, this cytokine plays a role in controlling the action of several cells involved in innate and acquired immunity. Regarding tuberculoid leprosy, the study reports a predominance of the cytokine IL-9. In addition, structures were visualized after staining, such as: granulomas formed by lymphocytes, others composed of macrophages, epithelioid cells surrounded by several lymphocytes. IL-9 was able to amplify the Th1 response, while being positively related to IFN-γ. In patients with the lepromatous form of leprosy, there was an increase in the amount of two cytokines, IL-4 and TGF-β. The data indicates that IL-9 acts by controlling the secretion of IL-4 and IL-10 in a negative way in both types of leprosy. A balanced relationship between IL-10 and TGF-β cytokines was evidenced, the synergistic action of these cytokines can positively mediate the expression of Th9 cells.
TARIQUE et al. (2017a) (49)/PUBMED/Experimental/JBI score 9/9	To study the role of IL-35 producing Tregs and Bregs in human leprosy.	Treg cells in individuals with both forms of leprosy (tuberculoid and lepromatous) than in healthy controls. In patients affected by leprosy, the secretion of IL-35 acted as an amplifier, significantly increasing the number of Treg CD4+CD25+ cells. The presence of IL-35 producing B cells was found in leprosy cases. It was verified that the levels of IL-35 were increasing as it passed from the tuberculoid point to the Lepromatous point, and that the expression of this cytokine was responsible for the Tregs and Bregs.
TARIQUE et al. (2017b) (50)/PUBMED/Experimental/JBI score 8/9	TCRγδ + FoxP3 + cells and their role during disease progression in leprosy patients.	The research identified the existence of CD4+TCRγδ+ cells, defined as CD4 + TCRγδ + FoxP3 +, in individuals affected by leprosy. These cells manifest the ability to suppress immune responses. People with the PB type of leprosy, with high serum titers of IFN-γ and IL-17, points out that these individuals can still stimulate the immune response, the same does not occur in people MB.
HUNGARY et al. (2017) (51)/PUBMED/Cohort/JBI score 11/11	To evaluate the potential to differentiate active PB leprosy from asymptomatic *M. leprae* (HHC) infection.	In individuals with the multibacillary form of leprosy (MB), high levels of antibodies directed to PGL-1 and LID-1 were detected, indicating its efficiency in identifying MB occurrences. Similar to IFN-γ quantification, CXCL10/IP-10 detection provided data enabling the distinction between PB and EC individuals, but it was not able to differentiate PB and HHC.
SAINI et al. (2017) (52)/PUBMED/Review/JBI score 10/11	Discuss the current scenario of T helper cells recently described in leprosy and its immunopathology to understand infection, T-cell anergy and new approach to treatments.	T cells have receptors related to CD4 and CD8, they are αβ, which is the most common chain, and γ/δ, both of which recognize *M. leprae* lipids. Cytokines expressed by Th1 are responsible for the rapid elimination of bacilli in lepromatous lesions. Th17 is a third subset of helper T cells, express IL-17 and in leprosy present the transcription factor RORC and STAT3. The presence of Th17 expressing IL-17A in skin lesions correlates more with the tuberculoid form. Furthermore, Th17 may confer protection to individuals who fail to elicit Th1 and Th2 responses.
SANTOS et al. (2017) (53)/PUBMED/Cohort/JBI score 10/11	To analyze, in a consolidated manner, the profile of inflammatory cytokines associated with the main and diverse clinical presentations of leprosy.	Regarding Th17 cells, it was verified that patients with the tuberculoid form of leprosy had a high number of CD4 + IL-17 + cells, the opposite was observed in those with the lepromatous form. This information shows that Th1 and Th17 responses act in a way to collaborate in the containment of *M. leprae.* Persons with the lepromatous form exhibiting serum levels of IFN-γ above 50 pg/mL had a higher risk of manifesting neurological deficit. Unlike the other groups, individuals with the MB form exhibited an elevated IFN-γ load.
TARIQUE et al. (2018) (54)/PUBMED/Review/JBI score 10/11	Highlight recent knowledge obtained on the role of different subtypes of T cells and their cytokines secreted during the progression of leprosy.	Treg cells act as suppressors of CD4+ T cells, are controlled by the transactional agent FoxP3 and divide into CD4+CD25+ Tregs and adaptive Tregs: Treg1 and Th3, IL-10 and TGF-β producers, respectively. After blocking IL-10 and TGF-β in patients with the lepromatous form, there was an increase in the functions of Th17 cells. Th17 cells produce IL-17, a cytokine classified as pro-inflammatory, responsible for increasing the expression of IL-6, IL-8/CXCL8, nitric oxide (NO), TNF-α and IL-1β, for several reasons. cellular species.
DE SOUSA et al. (2018) (55)/PUBMED/Cohort/JBI score 11/11	To investigate the IL-37 response in leprosy lesions.	IL-37 secretion by keratinocytes can act on dendritic cells, controlling their immune response model. Levels of IL-37 was higher in the tuberculoid type when compared to the lepromatous type. In addition to assisting in angiogenesis, the exacerbated production of IL-37 by endothelial cells can act in the form of immunosuppressive mechanisms. Regarding macrophages and lymphocytes, the amount of IL-37 expression was higher in the lepromatous type than in the tuberculoid type in macrophages, and for lymphocytes, the opposite was found. Thus, the predominance of IL-37 release by macrophages in the lepromatous form may be related to M2 macrophage reactions.
CHAVES et al. (2018) (56)/PUBMED/Cohort/JBI score 10/11	Identify and enumerate the Tregs in Leprosy, in order to understand the role of these cells.	The group of individuals with multibacillary leprosy (MB), when compared to the non-infected group (NI), showed a high amount of Tregs, as well as a high expression of FOXP3 in CD+ cells. In the group of people with paucibacillary leprosy (PB), in contrast to the MB group, a high fraction of D4+CD25+FOXP3+IL-10+ cells was detected, which ended up restricting not only bacillary multiplication as well as the spread of lesions. Both in the PB and in the MB groups there was a higher number of CD4+CD25+FOXP3+PD1+ cells than in the NI group, stating that the PD-1 pathway may drive the role of Tregs in individuals with leprosy.
SERRANO-COLL; ACEVEDO-SAENZ; CARDONA-CASTRO (2017) (57)/PUBMED/Review/JBI score 11/11	Notch signaling pathway and its relationship to TLRs, VDRs, NK cells, as well as its role in CD4+ T cell differentiation.	The types of TLRs involved in identifying *M. leprae* comprise TLR1/2, TLR2, TLR4 and TLR6. TLR1/2 activation leads to the production of IFN-γ, IL-6 and IL-12 that will play roles in the differentiation of *naive* T cells. Recognition of *M. leprae* peptidoglycans by TLR2 leads to cathelicidin synthesis, which in turn plays a critical role against mycobacteria. TLR4 signaling leads to expression of TNF-α, IL-6, IL-12, and IFN-1. TLR6 leads to lipid biogenesis, which causes an inhibitory effect on IL-12 and NO. After the TLR recognizes mycobacteria, the signaling process within the cell stimulates the appearance of Notch ligands on the cell surface, and the relationship between these two pathways may play an important role in the pathophysiology of leprosy. In NK cells we have the expression of immunoglobulin-like receptors (KIRs) that mediate activating and inhibitory signals of cell death, inhibition of Notch signaling decreased the activity of KIR activating genes.
OLDENBURG et al. (2018) (58)/PUBMED/Experimental/JBI score 9/9	Investigate and determine how PGLs interfere with the bactericidal functions of macrophages.	The macrophage TLR4 signaling pathway has its entirety affected due to the production of mycobacterial phenolic glycolipids (PGLs). Changes in the host’s bactericidal and inflammatory reactions are effects generated by PGL during chronic infection. It is indicated that PGLs influence interferon-β-inducing protein (TRIF), and that this does not depend on their action on TLR2 signaling. The ability of PGL to block nitric oxide (NO) production remained absent in MyD88 −/− macrophages, indicating that MyD88 is required to degrade TRIF, thus summarizing the operational importance of the reduction permeated by PGL-1 in TRIF for TLR4 signaling.
SADHU; MITRA (2018) (59)/PUBMED/Review/JBI score 10/11	To study the components of the adaptive immune system in leprosy patients.	The most relevant aspect of natural Killer cells is that, after their activation, the secretion of IL-4, TNF-α and IFN-γ, which are immunoregulatory cytokines, quickly occurs. Presenting a limited variety of TCR chains, invariant Natural Killer cells (iNKT), are capable of detecting glycolipid antigens of some bacteria when expressed by CD1d.
TOLEDO PINTO et al. (2018) (60)/PUBMED/Review/JBI score 11/11	Discuss seminal findings that reveal critical mechanisms of innate immunity and host metabolism with direct impact on disease outcome, where modulation may be the pathway to disease control.	TLR2/1 is a heterodimer responsible for identifying lipoproteins present in mycobacteria. The *S100A12* gene is mediated by TLR2/1 and encodes a protein effective in the direct elimination of *M. leprae.* NOD2, recognizing MDP, activates a pro-inflammatory response dependent on LRRK2, which ends up increasing the production of cytokines that will act on macrophages and their antimicrobial activities. Mycobacterial DNA extravasation into the cytosol triggers responses such as autophagy and type 1 IFN (IFN-I). The increase in MCP-1/CCL2 is responsible for contributing to the recruitment of monocytes, stimulates differentiation into M2 macrophages, and increases levels of prostaglandin E2 and IL-10.
KIM et al. (2018) (61)/PUBMED/Experimental/JBI score 9/9	To study the effects of physiological levels of 25D3 on differentiation, function and antimicrobial response of IL-15 MΦ against *M. leprae*.	The different phenotypes of IL-15 MΦ and IL-10 MΦ remained preserved during the process of monocyte differentiation, despite the disposition of calcitriol (25D3). However, the existence of 25D3 at the time of IL-15 MΦ differentiation led to high CAMP mRNA expression and cathelicidin protein levels. Vitamin D did not provide modifications in the phagocytic capacity of IL-15 MΦ, but it did confer a reduction in the bacterial load against *M. leprae*. Incorporation of 25D3 at the time of IL-15-driven differentiation of monocytes into macrophages enabled an enhanced stimulus of the vitamin D-dependent antimicrobial pathway, as well as an antimicrobial effect against *M. leprae* and a stimulus to cathelicidin.
DUA et al. (2019) (62)/PUBMED/Cohort/JBI score 10/11	To evaluate the role of Notch1 signaling in the regulation of T cell immunity in leprosy.	In cases of tuberculoid leprosy, a high percentage of Th cells with expression of the transmembrane receptor protein Notch1 was detected, the same was not observed in patients with lepromatous leprosy, where the percentage was lower. Therefore, it is indicative that Notch1 may play a role in disease control and in the regulation of the immune response during leprosy. Thus, demonstrating the role played by the Notch1 signaling pathway in the regulation of lymphoproliferation in the disease. Moreover, in cases referring to the lepromatous type, there was an increase in the production of IFN-γ by PGL-1 after activation of Notch signaling.
BEZERRA-SANTOS et al. (2018) (63)/PUBMED/Cohort/JBI score 11/11	To evaluate the immune response to crude and recombinant mycobacterial proteins ML2028 and the presence of multifunctional T cells expanded *in vitro* by this antigen.	The presence of ML2028-specific multifunctional CD8 T cells was evidenced in control samples and in healthy household contacts, however, this cell type is not identified as a determining factor for protection against *M. leprae.* In this study, IL-10 responses were not verified. The presence of high concentrations of IL-17A was found in the stimulation of antigens in the whole blood test in samples from healthy household contacts in multibacillary patients.
UPADHIAY et al. (2019) (64)/PUBMED/Cohort/JBI score 10/11	To study the effect of *M. leprae antigens* on STAT-4, STAT-6 and CREB transcription factors and their correlation with Th1/Th2 cell-mediated immune responses in leprosy.	The identification in peripheral blood mononuclear cells of the cytokines IFN-γ (Th1), IL-4 and IL-10 (Th2) corroborated the two types of responses, Th1 and Th2, related to the immunity of patients with leprosy. Healthy people and patients with tuberculoid leprosy had high baseline STAT-4 levels which corresponded to a considerable Th1 response in the subjects in question. In unstimulated PBMCs from leprosy patients, there was strong expression of STAT-6, which was not the case with cells from healthy people. Regarding the CREB transcription factor, there was a higher baseline expression in healthy people and patients with tuberculoid leprosy than in patients with lepromatous leprosy.
HOOIJ; GELUK (2021) (65)/PUBMED/Review/JBI score 10/11	Discuss the current state of knowledge about the cell immunity in response to *M. leprae*.	Monocytes differentiate into DCs in an IL-12 and IL-32 dependent manner, and subsequently T cells begin to produce IFN-γ to produce an effective Th1 response. In the peripheral blood of tuberculoid patients, there are more CD8+ T cells that express the cytotoxic proteins granulysin, perforin and granzyme B. The presence of (a particular subset of) CD8+ T cells is associated with the protective response and that these specific CD8+ T cells can be increased by IL-15 and antimicrobial mediated action. The three cytotoxic proteins serve as useful indicators both for reactive episodes and for an efficient antimicrobial response related to the tuberculoid leprosy subtype.
IDRISSI et al. (2017) (66)/PUBMED/Cohort/JBI score 11/11	To examine whether the deposition of complement activation products through Membrane Attack Complex (MAC) and C3d coincides with deposition of the *M. leprae* lipoarabinomannan (LAM) antigen.	The skin lesions of leprosy patients include complement, suggesting that inflammation caused by complement activation contributes to nerve damage in these patients’ lesions. When compared to individuals with tuberculoid disease, lepromatous patients had a much higher percentage of C3d deposition (relative to high concentrations of C3), MAC and LAM.
TELES et al. (2015) (67)/PUBMED/Cohort/JBI score 11/11	To investigate the association between IL-27 and the IFN-β-induced IL-10 pathway in immunosuppression of leprosy patients.	*M. leprae*-specific antibacterial activity was suppressed by IL-27. In skin lesions of individuals with progressive LL leprosy, IL-27 was more intensely expressed at the disease point and colocalized with macrophages that produced IFN-β and IL-10.

### Didactic illustrative schemes of the influence of the immune response against infection by *Mycobacterium leprae* with characterization based on the poles of severity of leprosy

**Figure 2:**
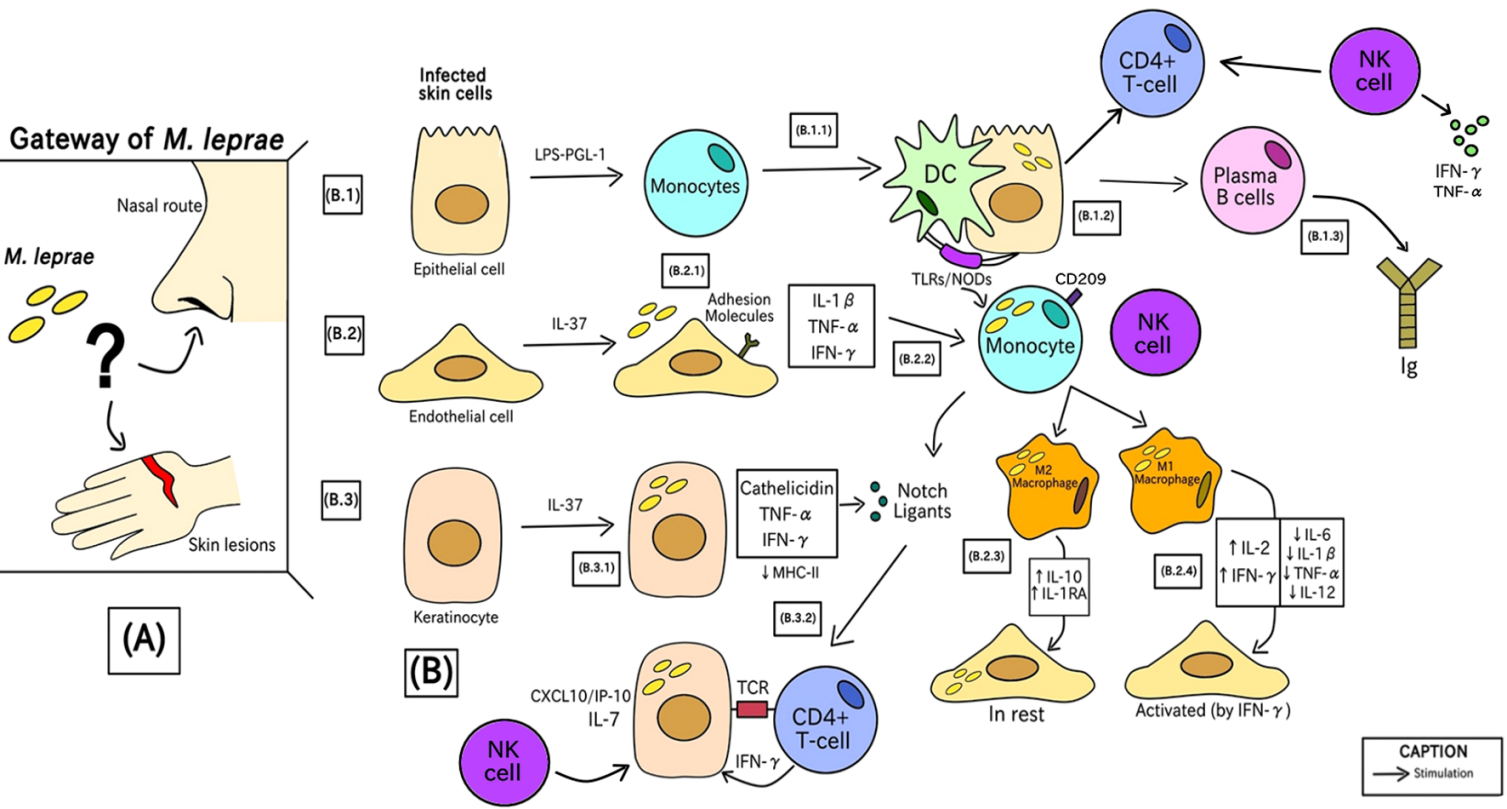
Phases of the generation of the human immune response against *M. leprae* since bacterial entry into the initial site of infection.

**Figure 2** demonstrates the immune responses induced by the infection of this pathogen. The chronological order of the steps is described by letters from A-B in parentheses.

In the first stage (A), bacterial entry into humans occurs through a still uncertain transmission route. The hypotheses behind this process involve the route of nasal epithelial cells or through keratinocytes and poorly differentiated endothelial cells due to minor injuries. Recently, the nasal route is the most accepted form of entry by the scientific community (1, 68, 69).

In (B) there is the characterization of cells infected in the first instance by *Mycobacterium leprae*. These cells are epithelial (B.1), endothelial (B.2), and keratinocytes (B.3) cells. These three cell types are the initial line of defense against *M. leprae*, and mammalian cell entry protein 1A (Mce1A) play a role in the cells’ ability to attract other cells (70–72).

(B.1.1) Epithelial cells, in contact with bacterial liposaccharide (LPS) and PGL-1 antigen, induce inflammatory mediators that can instruct monocytes to differentiate into mucosal dendritic cells (DCs) (73). (B.1.2) Through Pattern Recognition Receptors (PRRs), such as TLRs and NODs, DCs attach to the infected cell, process PAMPs from the bacteria and call for more monocytes (which can differentiate into other immune cells, such as macrophages, for example) (74, 75). (B.1.3) Next, there is the recruitment of B lymphocytes, responsible for the production and class switching of Immunoglobulins (Ig), acting as humoral immunity against infection, in addition to there being a Th1 response with cell-mediated immunity (CMI). Besides that, NK cells can be activated by cytokines produced during the initial immune response, such as IFN-γ, which is crucial in the defense against *M. leprae*, increasing antigen presentation of DC cells and T lymphocyte activation, strengthening the response against infection (57, 64).

(B.2.1) Endothelial cells stimulated by IL-37 expression through the secretion of inflammatory mediators, including IL-1β, TNF-α, and IFN-γ, which positively induce adhesion molecules like E-selectin, ICAM-1, VCAM- 1, and VLA-4 that allow immune cells to go to the infection site. This overexpression of molecules was not present in LL patients, suggesting that *M. leprae* influences this upregulation (55, 65). (B.2.2) Monocytes with high expression of CD209 are recruited and this cell signaling process also stimulates the induction of molecules called Notch Ligands (57). (B.2.3) Resting endothelial cells urge monocytes to differentiate into anti-inflammatory M2 macrophages, with elevated levels of IL-10 and IL-1RA, which do not generate an antimicrobial response (76). (B.2.4) IFN-γ–activated endothelium cells direct monocytes to develop into pro-inflammatory M1 macrophages, showing high levels of IL-2 and IFN-γ and lower concentrations of IL-6, IL-1β, IL-12 and TNF-α, which can limit bacterial growth. Patients with tuberculoid leprosy have active endothelial cells at the site of infection, which prompt M1 macrophages to provide an efficient antimicrobial response (47). After activation, NK cells release cytokines, such as TNF-α and IFN-γ, which not only promote the death of infected cells, but also stimulate other cells of the immune system, such as macrophages (77).

(B.3.1) Keratinocytes through the antimicrobial activity of IL-37 release MHC class II molecules to present *M. leprae* antigens by the release of cathelicidin, TNF-α and IFN-γ which stimulates the cellular immune signaling of Notch ligands (62). (B.3.2) Only lesions of patients with tuberculoid leprosy show these IFN-γ–induced keratinocytes (54). The interaction of the NK cell with the infected cell can also further increase the defense response with the activation of CD4+ T lymphocytes (78).

**Figure 3:**
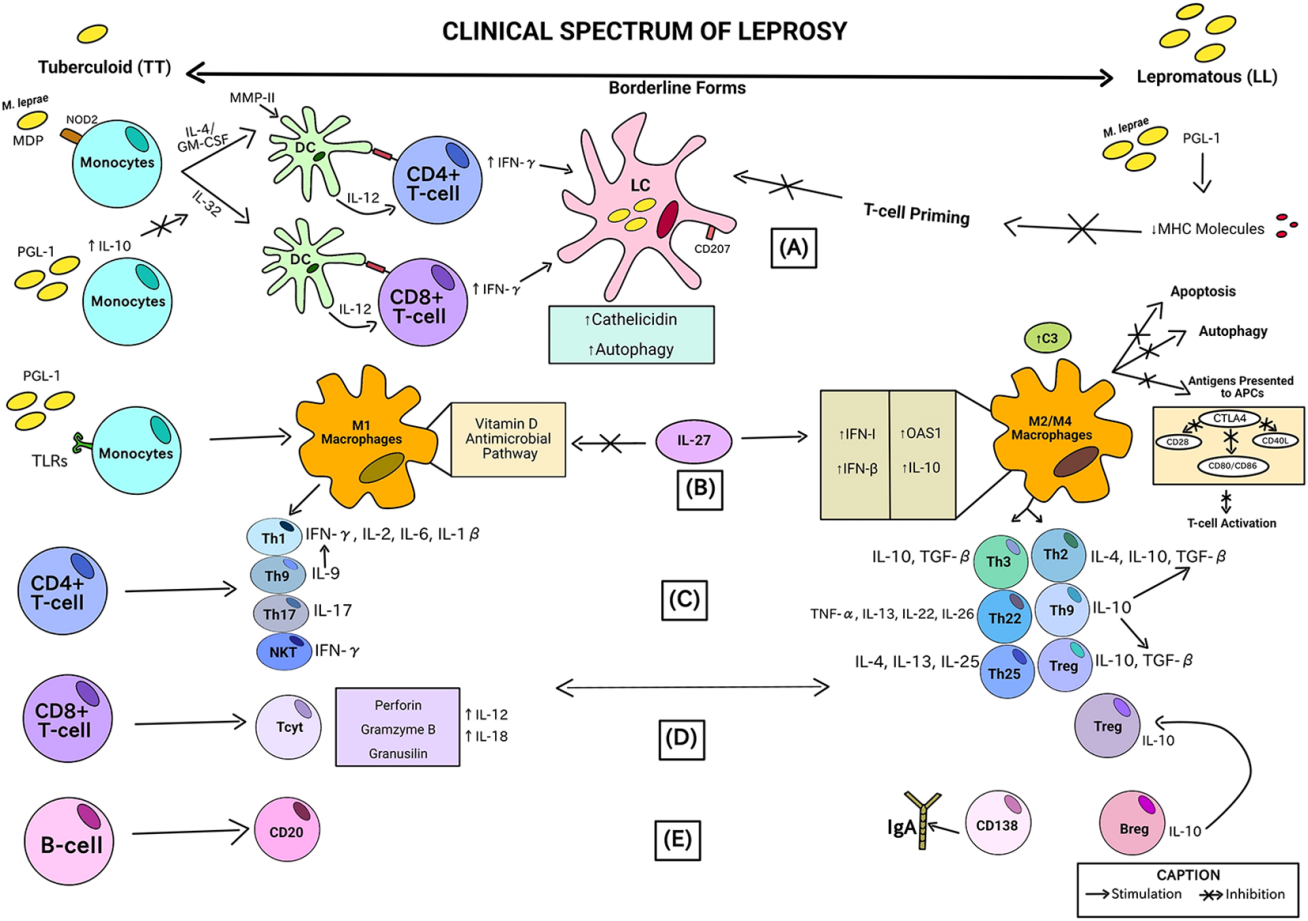
Association between immunopathological characteristics observed in the analyzes of the studies included in this review and the clinical forms of leprosy severity. This picture consists of a theoretical proposal for leprosy, based on individual data extracted from the studies included in this review, but which have not yet been proven in a joint and methodologically organized manner. No original study has been performed in clinically classified leprosy cases to prove such a clear differentiation using immunological parameters.

**Figure 3** is the visual identification of the summarized findings of this systematic review in relation to the association between observed immunopathological and the clinical forms of leprosy represented from left going to right like characteristics (according to the clinical spectrum of leprosy described by Ridley and Jopling classification). The chronological order of the steps is described by letters from A-E in parentheses.

In the initial stage (A), the innate immune response through monocytes can develop into dendritic cells (DCs) that act like APCs by a mechanism independent of IL-4/GM-CSF and IL-32. Muramyl dipeptides (MDP) from *M. leprae* are recognized by NOD2 and cause an increase in these pathways in monocytes. In the presence of IL-10 (induced by *M. leprae* PGL-1), the IL-32 pathway can be inhibited. Another *M. leprae* cell-wall antigen, MMP-II, activates CD4+ and CD8+ T lymphocytes via DC cells, stimulating the recruitment of adaptive immune cells through IL-12 induction, and producing IFN-γ. In lepromatous patients, the IL-12 production in response to *M. leprae* is poor. CD4+ T-cells and CD8+ T-cells receive antigens from DCs via MHC class II and class I, respectively. Less T-cell priming results from *M. leprae/*PGL-1 downregulating the production of MHC molecules. The skin or mucosa’s Langerhans cells (LCs) are the only cells that express langerin (CD207). When Langerhans cells infected with *M. leprae* are treated with IFN-γ, autophagy is induced and cathelicidin synthesis is increased. Cathelicidin is needed to activate the antimicrobial activity in these cells. *M. leprae* can be broken down and released, releasing antigens that can be presented to local T lymphocytes thanks to the enhanced autophagy in LCs (23, 31, 43).

In (B), monocytes exposed to *M. leprae/*PGL-1 in interaction with TLRs results in an increase in CXCL10/IP-10 and CCL2/MCP-1 production in M1 macrophages and a decrease in IL-10 and IL-1RA (M2/M4 macrophages) in Tuberculoid patients. Type I interferon genes, including IFN-β and OAS1, and IL-10, which are increased by IL-27, disrupt the vitamin D-dependent pathway, which is necessary for bacterial death in M1 macrophages. In lepromatous leprosy lesions, all these genes exhibit high levels of expression, which is consistent with the M2 macrophage phenotype, characterized by the high production of C3 (an element of the complement system). Antigens are not as readily available for T-cell antigen presentation when IL-10 levels are high because these processes are suppressed, along with autophagy and apoptosis. This lower availability of antigens to APCs is caused by the high release of the CTLA4 molecule, capable of inhibiting T cell receptors, such as CD28, CD40 ligand (CD40L) and CD86/CD80, generating less activation of T lymphocytes (52, 61).

In (C), activated CD4+ T cells can further differentiate by means of the type of phenotype of the recruited macrophage (M1 or M2/M4) into distinct T helper subpopulations according to the severity of the leprosy clinical form. In tuberculoid patients (TT), there is induction by M1 macrophages of Natural Killer T cell subpopulations - NKT (with high production of IFN-γ), Th17 (with high levels of IL-17), Th9 (with high production of IL-9) related to the release of IFN-γ by Th1 cells (subpopulation that produces IFN-γ, IL-2, IL-6, and IL-1β). On the other hand, in lepromatous patients (LL), there is secretion of subpopulations of T lymphocytes arising from the M2/M4 phenotype of macrophages referring to Th3 (with production of IL-10 and TGF-β), Th22 (elevated levels of TNF-α, IL-13, IL-22 and IL-26), Th25 (releasing IL-4, IL-13 and IL-25), Th2 (secreting IL-4, IL-10 and TGF-β), Treg (secreting IL-10 and TGF-β) and also Th9, but here it is related to the release of IL-10, a cytokine that is capable of inducing more TGF-β from both Th2 and Treg responses (18, 29, 49, 79, 80).

In (D), activated CD8+ T cells can differentiate into distinct subpopulations according to the severity of the leprosy clinical form. CD8+ cytotoxic T cells (Tcyt) are considered effectors and typical of patients with the TT form, as they produce potentially defensive proteins, such as perforins, gramzymes B and granusilins through IL-12 and IL-18 pathways. As regards the LL pole, there is an abundance of Treg-type CD8+ T cells, contributing to the T-cell anergy (81).

In the stage (E), in terms of B-cell differentiation, lepromatous individuals had more CD138+ cells, which is responsible for secreting high levels of IgA, although at the other end of the range, CD20+, a B-cell marker typically lacking on the terminally differentiated plasma cells, was more commonly seen. Patients with LL leprosy and, to a lesser extent, those with tuberculoid infection have been found to contain regulatory B-cells (Bregs) that produce IL-10. Bregs promote T effector cells to change into regulatory T cells concurrently as these cells expressed more FoxP3 and Programmed Cell Death Protein 1 (PD-1) (64, 82).

### Didactic illustrative scheme of the immunopathogenesis of leprosy

Leprosy is characterized by significant nerve involvement, with perineural inflammation being a hallmark of the disease. This inflammation may facilitate the entry of *M. leprae* into peripheral nerves via a vascular route. Once inside, the bacteria are ingested by Schwann cells, leading to various detrimental effects, including early axonal atrophy and eventual segmental demyelination of the affected nerves (83).

**Figure 4:**
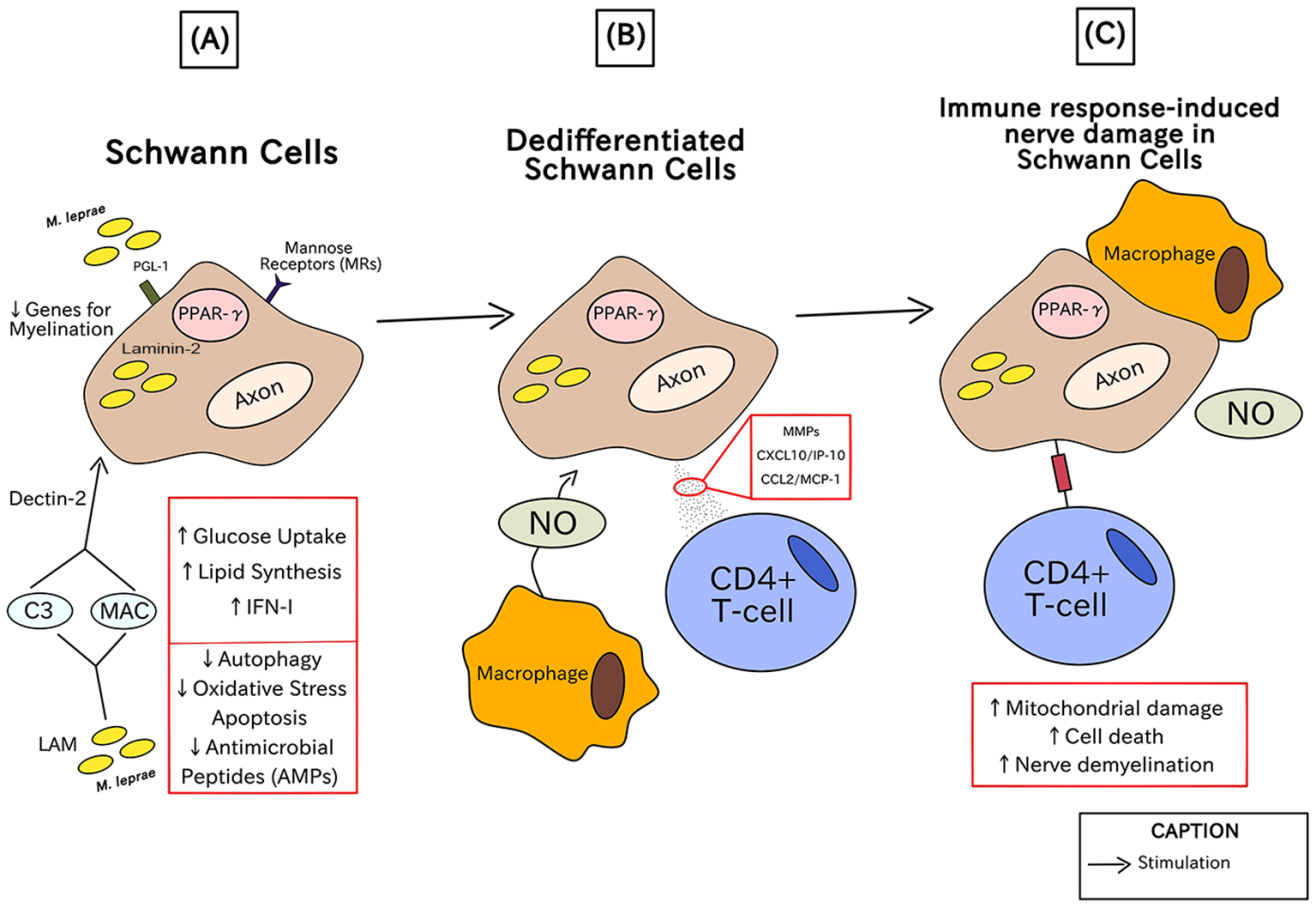
Immunopathogenesis of leprosy in the human nerves through injuries in Schwann cells.

In **Figure 4**, there is the characterization of the histological damage in human nerves caused by the negative immune response in patients with leprosy, particularly related to the most severe clinical conditions of leprosy. The chronological order of the steps is described by letters from A-C in parentheses.

In the initial phase (A), PGL-1 specific to *M. leprae* binds to laminin-2 on Schwann cells and is necessary for its internalization in Schwann cells where it causes the upregulation of mannose receptors (MRs), such as CD206, via peroxisome proliferator-activated receptor gamma (PPAR-γ). Simultaneously, the interaction between the glycolipid lipoarabinomannan (LAM), one of the components of the most external part of the mycobacteria, which acts like a kind of PAMP and activates the Schwann cell by the formation of opsonin C3 and Membrane Attack Complex – MAC, and the cellular receptor Dectin-2, responsible for β-glucan recognition implicated in the phagocytic process. To survive in Schwann cells, *M. leprae* induces the production of IFN-I-related genes, lipid synthesis, and regulates glucose absorption, whereas it downregulates oxidative stress apoptosis, autophagy, and the expression of antimicrobial peptides (AMPs). Through shrinking the expression of genes necessary for myelination, such as Krox-20, myelin basic protein (MBP), and myelin protein zero (MPZ), it results in dedifferentiated Schwann cells (20).

In (B), Dedifferentiated Schwann cells generate matrix metalloproteinases (MMPs), CXCL10/IP-10, and CCL2/MCP-1 (which are chemoattractants of macrophages and CD4+ T cells) and are very prone to invasion. Nitric oxide (NO) is produced by macrophages in response to *M. leprae* PGL-1 via CXCL10/IP-10 and CCL2/MCP-1 (76).

The final stage (C) is characterized by the subsequent mitochondrial damage in axons that initiates nerve demyelination and can cause cell death. Along with the activities of macrophages and NO, antigen presentation via CD4+ T-cells results in the death of infected Schwann cells. These factors contribute to the nerve damage and subsequent pathological clinical conditions in leprosy (84).

### Innate immunity in leprosy

It is known that the *M. leprae bacillus* resides in macrophages, dendritic cells and Schwann cells and penetrates the cells through the action of a component of the bacterial cell wall, called glycolipid-phenolic-1 (PGL-1), which binds to that of complement C3 through complement receptors CR1, CR3 and CR4 (13, 18). Doz-Deblauwe et al. (2019) identified in mice infected with *M. leprae* that the binding of PGL-1 to CR3 increased bacterial phagocytosis by polymorphonuclear neutrophils (PMNs) and in lung macrophages, in addition to increasing bacterial invasion in dendritic cells (DCs), increasing the selective production of IL-2 by these cells and IL-10 by macrophages. Study data indicate that by targeting CR3, PGL-1 triggers the Syk/calcineurin/NFATc pathway that rewires the innate immune response in three key innate cells. rBCG: PGL-1 allows effective uptake by PMNs and lung macrophages if CR3 is present and Syk is active. Lung cells infected by rBCG: PGL-1 produce high levels of IL-10 when CR3 was present (20).

The presence of NK cells is indicative of a robust immune response, which may help limit the spread of the bacillus. In cases of lepromatous leprosy, where there is a depression of the cellular immune response, NK cell activity may be insufficient to control the infection, resulting in more severe manifestations of the disease (85).

Furthermore, studies identified that in the MB form of leprosy, high levels of antibodies and IFN-y directed by PGL-1 were present, showing the efficiency of the immune system in identifying this antigen (51, 62). The results showed that IFN-γ induces the autophagy-dependent antimicrobial activity of Langerhans Cells - LC against cutaneous pathogens, such as *S. aureus*, *S. pyogenes* and *C. albicans*. Autophagy in leprosy contributes to an antimicrobial response in CL, which allows DC to process pathogen-derived antigens to T cells and direct the adaptive response of T cells (23).

Monocytes that differentiate into DC in the presence of PGL-1 and bacterial LPS demonstrate immunosuppression with respect to the production of inflammatory cytokines and immune regulatory molecules. Considering that DCs can produce the PGL-1 antigen, masking it enhanced the DC-mediated T cell response, demonstrating that PGL-1 presentation dampens the T cell response. Activation of DCs can be detected by increased expression of MHC class I and II molecules, CD86 and CD83 when DCs are stimulated by pure *M. leprae* matrix metalloproteinase (MMP)-II (through TLR signaling). These MMP-II pulsed DCs trigger T cell IL-12 and IFN-γ production, resulting in an efficient Th1 response (73). High amounts of IL-10 prevent DC development via NOD2/IL-32 in lepromatous patients’ monocytes, but recombinant IL-32 added *in vitro* restored DC differentiation in these individuals.

IL-32-induced DCs are crucial to the host’s defense against *M. leprae* (74).The activity of macrophages involves the production of oxygen species and reactive nitrogen, through the complex NADPH-oxidase and nitric oxide (NO) (75). Oldenburg et al. (2018) demonstrated a limited ability of macrophages to induce nitric oxide synthase and generate NO to fight bacterial infection (58).

Toll-like receptors (TLRs) are present on macrophages, DCs and Langerhans cells, and mainly TLR1/2, TLR2, TLR4 and TLR6 are involved in the recognition of *M. leprae* PAMPs (57). The TLR1/TLR2 heterodimers are known to be responsible for recognizing mycobacterial lipoproteins and activating the pro-inflammatory response. Krutzik et al. (2003) identified that TLR1 and TLR2 are expressed in greater amounts in individuals with tuberculoid leprosy than in patients with lepromatous leprosy (86).

Polycarpou et al. (2016) found that this bacillus has a ligand that activates TLR4 leading to signal transduction and is present in human macrophages, the preferred environment for *M. leprae.* Furthermore, it was identified that the use of a neutralizing monoclonal antibody against TLR4 influenced the decrease in the production of pro-inflammatory cytokines, such as TNF-α, CXCL10/IP-10 and IL-6. The CD16, a signaling marker for immune cell recruitment, acts by mediating the TLR4 response. Furthermore, it has been shown that PGL affects the TLR4 signaling pathway, increasing the exposure of macrophages to PGL and leading to a reduction in the levels of the interferon-β inducing protein (TRIF), containing the TIR domain, resulting in a low production of INF-β and CXCL10/IP-10 (47).

Monocytes in the presence of galectin-3 have a reduced antigen presentation, indicating an influence of the lectin through glucose uptake on the low immune response in leprosy patients (33). GM-CSF-derived APCs stimulate CD1B-restricted T cells to proliferate in response to antigen. Macrophages are generally classified into M1 and M2, according to the type of Th response (Th1 or Th2), and M2 macrophages are directly associated with the Th2 response present in LL leprosy (52). M2 macrophages from patients who have the disease in a disseminated form present co-stimulation of the expression of molecules such as: CD68 and CD163, in addition to the anti-inflammatory cytokines IL-10 and TGF-β (31). Other types of macrophages were also disregarded, such as the M4 phenotype, which is more highly expressed in lepromatous leprosy patients and may indicate that this subpopulation is less successful in eradicating the bacillus, favoring disseminated presentations (87).

The bacterial death in M1 macrophages depends on the vitamin D-dependent pathway. In macrophages infected with *M. leprae*, this response is innately active, but it is inhibited by *M. leprae* ‘s production of type I IFN. CYP27B1, which transforms the inactive prohormone substrate 25-hydroxyvitamin D (25D) into the active vitamin D hormone 1,25-dihydroxyvitamin D, is necessary for the antimicrobial response, was blocked by the generation of IFN-β and OAS1 in infected monocytes/macrophages *in vitro* (67, 76, 88).

The M2 phenotype is advantageous for *M. leprae* to survive in macrophages. This is consistent with the relationship between the *in vitro* distorting of macrophages infected with *M. leprae* toward the M2 phenotype and the suppression of autophagy and apoptosis. This inhibition of macrophages can stop T cells from receiving an effective antigen presentation. Uninfected macrophages were able to deliver antigen by consuming apoptotic cells, while infected macrophages were not effectively engulfed (89). The negative feedback loop that caused the suppression of autophagy is as follows: Initially, live *M. leprae* increased macrophage autophagy, which resulted in decreased pro-inflammatory cytokine expression. These macrophages suppressed further activation of autophagy by selectively priming anti-inflammatory T cells that produced high amounts of IL-10 (62, 64).

Leprous neuropathy was shown to have downregulated expression of multiple mitochondrial genes when compared to non-leprous neuropathy in nerve lesions from individuals with peripheral neuropathy (65). Then, MB and PB patients can be distinguished using indicators of mitochondrial dysfunction, which have also been shown in other neurodegenerative illnesses (90).

*M. leprae* controls the autophagy mechanism of human monocytes to create a safe space inside cells for bacterial replication. The survival of *M. leprae* is not influenced by the lack of L-tryptophan, and this is due to the activation of the IDO-1 signaling pathway dependent on IL-10 and iron (Fe). *M. leprae* uses glucose transporter 1 to enhance glucose uptake in infected cells. Live bacilli, during *M. leprae* infection, prevent the formation of free radicals by activating carbons in the electron transport chain for the formation of lipids (60).

In this sense, in leprosy patients, lipid metabolism is also impacted. High-density lipoprotein (HDL) becomes dysfunctional because of the oxidation of ApoA1, which is a major HDL protein and impairs macrophages’ ability to remove cholesterol (65). The presence of foamy macrophages, which are typical of lepromatous leprosy, actively contributes to *M. leprae* ‘s improved survival in the host; hence, it is not unexpected that these patients’ lesions tend to have upregulated lipid metabolism-related genes (90). While HDL from healthy people retained DC function, defective HDL in these patients also led to a reduction in the clearance of oxidized lipids, which in turn inhibited the DCs’ ability to deliver antigen to T-cells. Therefore, increased ApoA1 production in response to delayed neuronal damage (91).

### Adaptive immunity in leprosy

The recognized antigens can recruit T lymphocytes through CD4+ T Cell Receptors (TCR). In turn, keratinocytes secrete CXCL10/IP-10 and IL-7 because of CD4+ Th-cells producing IFN-γ. There are several categories for which naive T cells can differentiate, such as Th1, Th2, Th3, Th9, Th17, Th22 and Th25, and for this differentiation to occur there is the involvement of Notch. Therefore, it is possible to point out which individuals are vulnerable to producing anergic forms of leprosy due to the Notch ligands demonstrated in APC cells of *M. leprae* carriers (57). Cytotoxic CD8 T-cells mediated by NK cells activity can directly kill infected cells, among others via the secretion of IFN-γ and TNF-α or the release of cytotoxic granules and their frequency is higher in tuberculoid patients than the lepromatous form. The first illness for which CD8 suppressor T cells cloned from a patient with borderline lepromatous disease were discovered was leprosy. Depending on the function of the CD8 T‐cells they are either associated with PB leprosy (cytotoxic - Tcyt) or MB leprosy (Tregs) (81).

It is known that Th1 and Th2 play an important role in the immune response against mycobacteria. IL-4 levels, produced by Th2, were significantly higher in LL patients than even in healthy subjects and in tuberculoid patients (64). IL-10 is an anti-inflammatory and immunosuppressive cytokine produced by T cells and macrophages, whose overactivation decreases lymphocyte-induced immunity and is present in lesions of MB patients (54).

However, contrary to most of the literature, the work carried out by Kumar et al. (2011), one of the studies included in this review sought to analyze this immunological relationship between Th1 and Th2 and demonstrated that the values of IL-4 and IL-13, cytokines associated with Th2, had their levels unchanged in BL/LL, with no correlation with the bacteriological index. Th3 immune responses (in the absence of Th1 and Th2) allow *M. leprae* to progress. It deduces that through raising the expression of Cbl-b, a member of mammalian Cbl family proteins (ubiquitin ligases thought to negatively regulate TCR signaling), the overexpression of TGF-β and CTLA4 causes T cell hyporesponsiveness, a significant leprosy characteristic (29).

The Th17 response, responsible for producing the cytokine IL-17 and which has a RORC and STAT3 transcription factor. This type of response plays a protective role in individuals who lack the ability to present Th1-type immune responses (37). Increased production of IL-17 in tuberculoid leprosy was related to greater recruitment of inflammatory cells, increased activation of endothelial cells and help in maintaining the chronic inflammatory process (18).

Other types of T cells have essential roles in the formation of host immunity, such as natural killer T cells (NKT) and regulatory T cells (Tregs). These cells recognize mycobacterial antigens that are presented by CD1, which may reduce IL-4 production and increase IFN-γ production, increasing the potential for bacilli elimination (59). In cases where NKT cells may present a late development, the cells produce a low level of IFN-γ, causing non-differentiation of CD4+ T cells in the Th1 phenotype, helping in the development of the LL form of the disease (57).

The development of the Treg and Th17 lineages is connected by the fact that TGF-β also induces RORγt, a key transcription factor in Th17 cell differentiation. FOXP3 can decrease RORγt activity and promote Treg development in the absence of a second signal from a proinflammatory cytokine. However, FOXP3 activity is suppressed and the Th17 differentiation pathway is triggered when the cell also gets a signal from a pro-inflammation cytokine (for example, IL-6) (45).

Tregs in leprosy are in greater quantity in individuals with the lepromatous type. It is considered that the circulation of Treg cells to the sites of lepromatous lesions is related to the presence of tissue chemokines produced at the site of the lesion. Tregs use PD-1 ligands to block B cell activation directly, which prevents the generation of antibodies, hampers B cell growth, and triggers B cell death. In the same way, the regulation of key genes linked to the phenotype of Treg cells is controlled by a vast protein complex that FOXP3 is one of its components (37).

FoxP3-positive Treg cells act by reducing the activity of effector T cells, such as Th17 cells, and thereby negatively controlling the immune response within the host during intracellular infections. Th17 cells are helper T cells that act by increasing the number of Th1 effector cells, in the recruitment of neutrophils and in the activation of macrophages. Secretion of IL-10 by Treg cells in individuals with the lepromatous form is considerably associated with polarized immunity distinct to small IL-17 by CD4+ T cells of the cluster itself (59).

To survive, Hansen’s bacillus can induce TCRγδ + FoxP3 + immunosuppressive cells, through the control of phosphoantigens. A small proportion of T cells possess the TCRγ/δ complex, whereas most T cells possess the αβ complex. γ/δ TCRs identify phosphoantigens present in mycobacteria without depending on the major histocompatibility complex - MHC. A high amount of γ/δ T cells in the skin and blood has been associated with granulomatous reactions in leprosy people of the LL (54).

NKT cells, which correspond to innate lymphocytes and share surface characteristics with NK cells, are thought to be diminished in leprosy patients. Once triggered by antigen recognition, NKT cells can release a variety of pro-inflammatory mediators quickly. However, NKT cell numbers grew following stimulation with PGL-1 or mannose-capped LAM, particularly in tuberculoid individuals (with more IFN-γ) (79).

Th9 lymphocytes are associated with the pole tuberculoid and arise from Th0 cells, which in the presence of IL-4 and TGF-β differentiate to Th9, producing cytokines such as IL-9, IL-10, and IL-21. The IL-9 cytokine is directly involved with the regulatory activities of cells involved in innate and adaptive immunity. SOUSA et al. (2016) observed the predominance of IL-9 in TT individuals, mainly in lymphocytic granulomas and granulomas composed of macrophages. It has been shown that an increase in IL-10 caused by Th9 activation in tissue lesions of LL patients inhibits macrophage activation and adversely controls the production of IL-12, IFN-γ, and TNF-α. Furthermore, it was verified that IL-9 had the blocking effect of IL-4 and IL-10, being able to positively regulate the expression of Th9 lymphocytes (48).

Th22 cells are recognized for being part of a group of CD4+ T cells that are responsible for the production of isoforms belonging to the Fibroblast Growth Factor (FGF) family and cytokines such as TNF-α, IL-13, IL-22, and IL-26. These cytokines by Th22 subpopulation, such as Th2 cells, are linked to humoral responses and the activation of tissue regeneration processes, leading to a clinically less potent immune response to intracellular infections (80).

Once regards to the Th25 response in leprosy, it appears to be closely linked to the anti-inflammatory pattern in the LL disease mode, i.e., promoting tissue repair or immunosuppression. It is well established that the cytokines IL-4 and IL-13 have a direct role in the Th2 lymphocyte response and M2 macrophage development. Furthermore, IL-4 and IL-13 control the humoral immune response and B cell activation. IL-25/IL-17E is a member of the IL-17 cytokine family and, unlike IL-17A and IL-17F, inhibits the development of both Th1 and Th17 cytokines (92).

Moreover, B-cells is involved in disease etiology in lepromatous patients and related to the inverted gradient between CD138- and CD20-positive phenotype by B cells from the tuberculoid to the lepromatous pole. Patients with lepromatous disease and, to a lesser extent, those with tuberculoid disease have regulatory B-cells (Bregs) in the blood that produce IL-10 (22). Bregs promote T effector cells to change into regulatory T cells while these cells expressed more FoxP3 and PD-1. This B-cell fraction led to the creation of the immunosuppressive IL-10 and may play a significant role in *M. leprae* maintenance (82).

In addition to chemokines, epithelial cells actively control the local immune response. The discovery of IgA antibodies against entire *M. leprae* in saliva suggested that mucosal immunity may play a protective role in responses to *M. leprae* infection. Contacts of untreated leprosy patients, who are often exposed to *M. leprae*, had greater salivary IgA levels than endemic controls, indicating an active mucosal immune response. Additionally, high salivary IgA levels were seen in household contacts, particularly in those who often interacted with MB patients, in response to LAM or PGL-1 compared to controls. Therefore, it appears that the IgA levels in saliva correspond with the degree of exposure to *M. leprae* (93).

## Discussion

This article presents a comprehensive overview of the immunopathological aspects of leprosy, focusing on the relationship between the immune response and the severity of the disease caused by *Mycobacterium leprae*. The study employs a systematic review methodology to synthesize findings from various studies published between 2011 and 2022. This review highlights a significant correlation between the severity of leprosy and the immune response.

In order to survive while immunologically hidden within host cells, the bacteria may need to evade immune surveillance systems, allowing long-term parasitization (94). By boosting inducible nitric oxide synthase (iNOS) in the host macrophages, *M. leprae* ‘s adherence and invasion of Schwann cells encourage brain injury. When the bacterial PGN is recognized, the nucleotide-binding oligomerization domain (NOD)-like receptors (NLRs) start an immunological response (74).

Defense against the *M. leprae* bacillus is initiated by the host’s innate immune response, followed by the adaptive immune response (41). Innate immune cells act such as APCs, like macrophages and dendritic cells (DCs) can be activated by the functional expression of pattern recognition receptors by epithelial cells, including Toll-like Receptors (TLRs). According to the traditional Th response (Th1/Th2), macrophages may be divided into two categories, M1 and M2. However, the traditional Th1/Th2 antagonistic response pattern associated with the prognosis of Hansen’s disease has recently come under scrutiny due to the wide range of immune response data reported by new studies linked to other T helper subpopulations that differ in the disease’s classic symptomatology (18).

It was reported here that lower release of MHC molecules in response to phenolic glycolipid-1 (PGL-1) is associated with more severe forms of the disease (20, 95). This suggests that a robust immune response, characterized by effective antigen presentation, is crucial for controlling the infection. The differentiation of macrophages into distinct phenotypes plays a critical role in the immune response to leprosy (88, 89). M1 macrophages, associated with the Th1 response, are linked to the tuberculoid form of leprosy, while M2 macrophages, associated with the Th2 response, are more prevalent in lepromatous leprosy (47). This polarization affects the overall immune landscape and the body’s ability to combat the infection.

Activated CD8+ T cells exhibit different profiles depending on the clinical form of leprosy. The review notes that in severe cases, there is a specific induction of regulatory T cells (Tregs), which contribute to T cell anergy, further complicating the immune response (49, 82). The findings emphasize the importance of the innate immune system in the initial presentation of leprosy, while the adaptive immune system is more influential in the progression of nerve damage and the clinical manifestation of the disease (21, 81).

Numerous reviews have already been conducted on the topic of immunology in leprosy (13, 41, 52, 57, 59, 60, 65). This is due to several factors, such as being an ancient disease and with recorded data present in the literature for a long time, the need to associate different complex data and because it is a public health problem. However, it is necessary and important that there are updated reviews on the subject since many new immunological discoveries in leprosy have been made in the scientific community in recent years and are dispersed in the literary environment.

When compared to other literature on the immunological aspects of leprosy, such as the studies by Froes et al. (2022) and Karamova et al. (2020), the findings of this present review align with the understanding that distinct immune responses are critical in determining the clinical outcomes of leprosy (18, 19). For instance, Froes et al. (2022) discuss the roles of various T cell subsets and cytokines in different clinical presentations, reinforcing the notion that the balance between Th1 and Th2 responses is pivotal (18). Additionally, this review’s emphasis on macrophage polarization and its impact on disease severity resonates with findings from other studies, such as those by Dang et al. (2019), which highlights the role of Langerhans cells in antimicrobial responses and antigen presentation (23).

Molecular epidemiology studies with *M. leprae* in humans and primates are important for identifying genetic profiles and, through this, understanding the origin, transmission pattern and concentration of bacterial strains related to leprosy events. In particular, the geographical restriction of *M. leprae* strains refers to the specific geographical distribution of the different strains of *Mycobacterium leprae* around the globe (96, 97).

This geographical restriction consists of a theory under investigation that has not yet been proven, but is under investigation. It is supposed that it can be observed in different regions of the world, influencing the incidence and prevalence of the disease. Multibacillary leprosy (LL form) is more common in areas like Saudi Arabia, while paucibacillary disease (TT form) is more frequent in localities such as Brazil (98–100).

Moreover, evidence suggests that differences in the genome of the *M. leprae* strain may be able to distinguish between distinct clinical manifestations of leprosy, in addition to the host response, and to cause differential gene expression in human macrophages. According to analysis of the samples in India, TTC repeats are prevalent in Indian LL skin samples, which set them apart from other *M. leprae* strains found in the country (101). The genotypic difference is related to the spread and transmission of leprosy and related to disease types (102).

Specific genotypes of *M. leprae* were also linked to a particular clinical pole. Genotype 1 of *M. leprae*, linked to European geographic origins, has been associated with the tuberculoid pole (TT form), while genotype 2, linked to Brazilian regions such as Pernambuco and Ceará, has been related to the lepromatous pole (LL) of leprosy (103–106). Furthermore, there is evidence of the contribution of the specific characteristics of the strain linked to the leprosy event in the regulation/modulation of clinical states. An example of this is the evidence that leprosy cases caused by *Mycobacterium lepromatosis* were associated with diffuse lepromatous leprosy (DLL), although it should be mentioned that the population size screened in this kind of papers for the prevalence are too small to conclude (107, 108).

Regarding the future perspectives, the condensation of these information of this present article can help scientists and health professionals to see answers to immunopathogenic gaps, generate new hypotheses for the progression of leprosy, new ways of targeting to fight the disease, as well as new questions for both *Mycobacterium leprae* as well as other infections. In addition, clarification of the pathogenic interface of interaction between bacterial agent and host can help in the search for diagnostic and prognostic biomarkers for leprosy. In this sense, it is necessary to carry out more studies on the complete proteome and transcriptome profile of leprosy patients and household contacts with the disease.

The limitations of this study come up against the following conditions: a) the case definition of a leprosy patient according to the different classifications of the disease in each study; b) different forms of diagnosis; c) different strains of *M. leprae*; d) different genotypes of *M. leprae*. In order to find novel solutions to issues about the immunological aspects of leprosy, further study must be done along methodological lines. This includes investigating which speculations of biological elements, in particular, raise the likelihood that an individual would become lepromatous and erode their defenses: (1) examination of experimental research on the immunoregulation of the cell-mediated immune response in relation to nonreplicating antigens; (2) investigation of immune responses and their regulation in leprosy in mice; (3) evaluation of the outcomes of human immunotherapy trials; and (4) meticulous scrutiny of immune responses in other persistent infections in humans.

## Conclusions

The progression factors of leprosy are linked to several interconnected complex molecular of host-parasite interaction. The early development of the host infection depends on these cells’ capacity to deliver antigens and control the polarization of macrophages. Apoptosis and autophagy may be possible with differing phenotypes of the macrophages that have been attracted to the infection site, which may result in a biphasic response, i.e., allowing replication and further dissemination or its elimination.

Innate immune cells try to reduce the bacterial burden by activating the adaptive immune system. The capacity of antigen presentation is actively downregulated by *M. leprae* as the bacterial load rises, which lowers the activation of T cell-mediated immunity (TT or LL form). As a result, the innate immune system plays a far larger role in the initiation of nerve damage, which is a symptom of the illness’s first presentation, and in many key immunological reactions, such as inflammation and the removal of dead *M. leprae*, even while the adaptive immune system intensifies nerve damage and determines the kind of leprosy.

## Data Availability

The original contributions presented in the study are included in the article/Supplementary Material. Further inquiries can be directed to the corresponding author.
